# Supplementary figures and images for: COVID-19 induces a hyperactive phenotype in circulating platelets
Source: PLoS Biol. 2021 Feb 17;19(2):e3001109. doi: 10.1371/journal.pbio.3001109 (PMC7920383; doi:10.1371/journal.pbio.3001109)

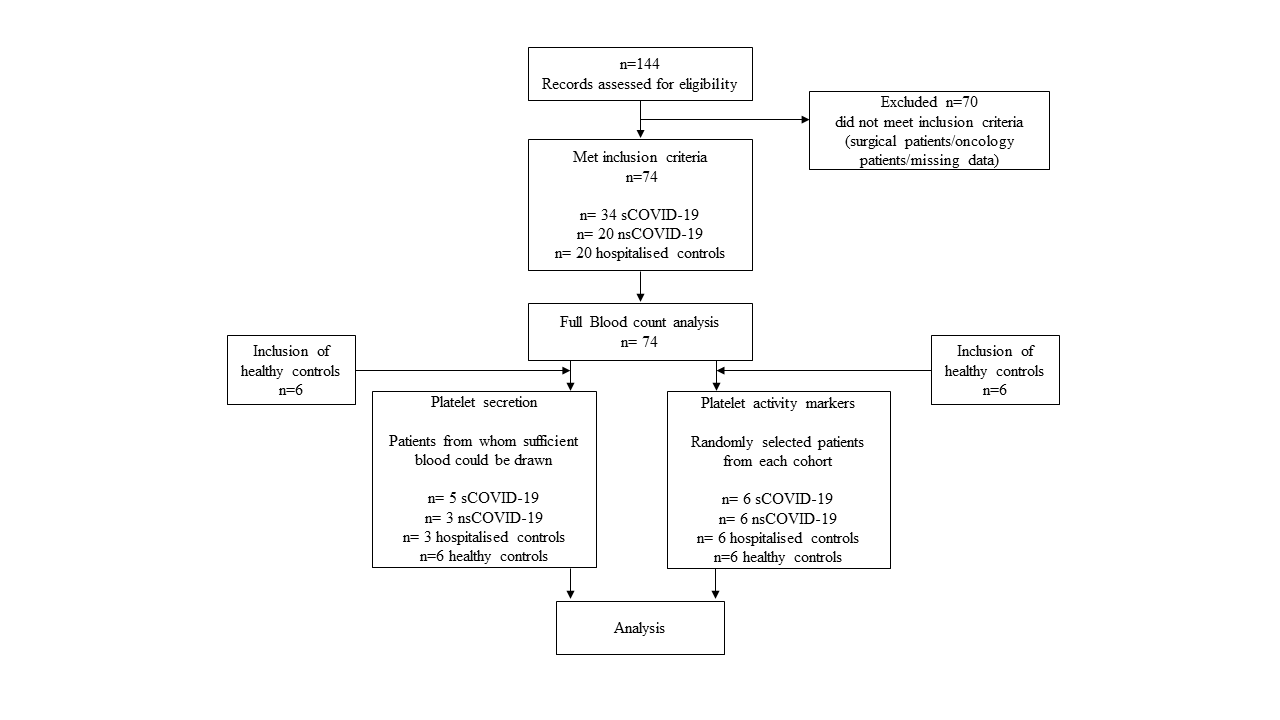

Supplement: S1 Fig — nsCOVID-19, nonsevere COVID-19; sCOVID-19, severe COVID-19. (TIF) [file pbio.3001109.s001.tif]

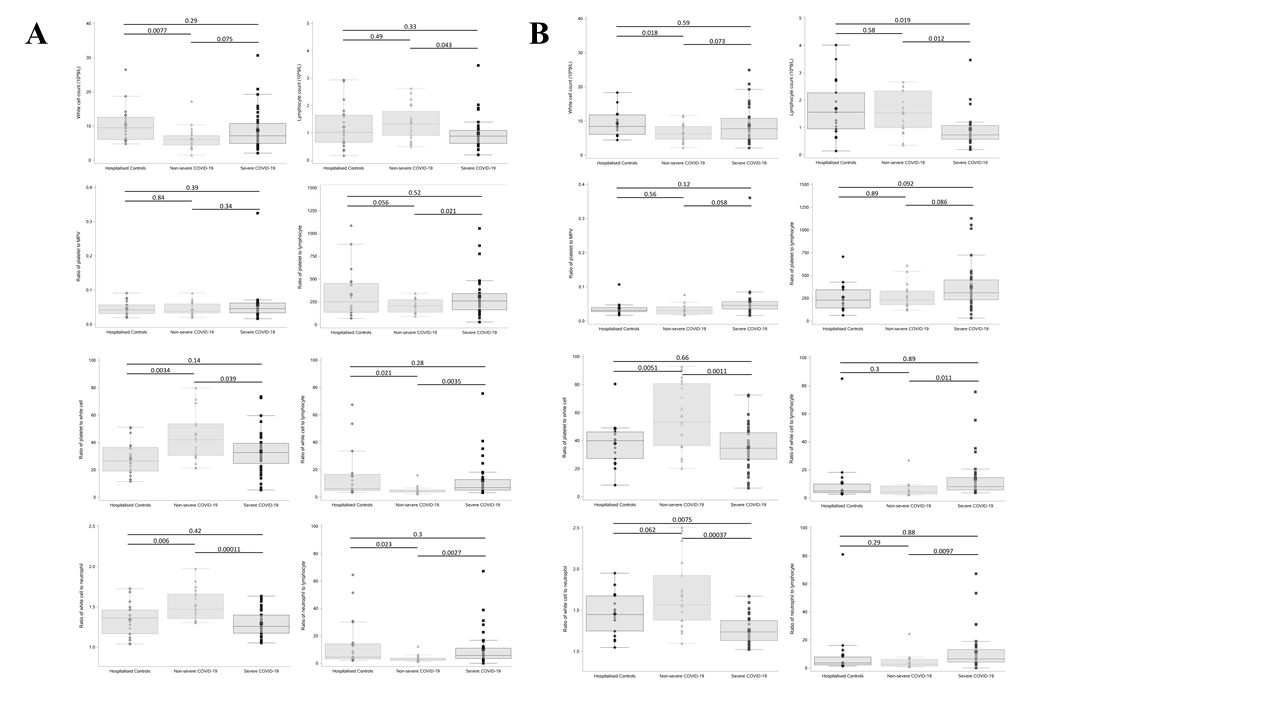

Supplement: S2 Fig — White cell and lymphocyte counts as well as platelet-to-MPV, platelet-to-lymphocyte, platelet-to-white cell, white cell-to-lymphocyte, white cell-to-lymphocyte, and neutrophil-to-lymphocyte ratios on day of hospitalisation (A) and 7-day postadmission (hospitalised controls and nonsevere COVID-19) or day of transfer to ICU (severe COVID-19) (B) (see individual data in S1 Data). Boxplots represent the data median (line inside the box) and the IQR (outline of the box) together with data maximum and data minimum (whiskers) and individual observations. COVID-19, Coronavirus Disease 2019; FBC, full blood count; ICU, intensive care unit; IQR, interquartile range; MPV, mean platelet volume. (TIF) [file pbio.3001109.s002.TIF]
