# Supplementary material for: COVID-19 induces a hyperactive phenotype in circulating platelets
Source: PLoS Biol. 2021 Feb 17;19(2):e3001109. doi: 10.1371/journal.pbio.3001109 (PMC7920383; doi:10.1371/journal.pbio.3001109)
Supplement: S1 Table — Results are presented as mean ± SD. (DOCX) [file pbio.3001109.s003.docx]

**S1 Table:** Clinical characteristics of severe and non-severe patients with confirmed COVID-19, hospitalised controls without COVID-19 and healthy controls. Results are presented as mean±SD.

|  | **Severe COVID19 (n=6)** | **Non-severe COVID19 (n=6)** | **Hospitalised Controls (n=7)** | **Healthy Controls (n=6)** | **p-value** |
| --- | --- | --- | --- | --- | --- |
| Age (years) | 57.0 ± 5.5 | 67.0 ± 20.7 | 61.3 ± 11.28 | 30.8 ± 6.5 | **0.0010** |
| BMI (kg/m^2^) | 26.58 ± 4.79 | 22.93 ± 1.25 | 33.98 ± 15.32 | 25.86 ± 4.37 | 0.594 |
| Male, n (%) | 5 (83%) | 4 (67%) | 4 (57%) | 3 (50%) | 0.819 |
| White blood cells (10^9^/L) | 9.41 ± 2.46 | 4.85 ± 0.99 | 8.67 ± 2.54 | 6.03 ± 1.35 | **0.0007** |
| Red blood cells (10^12^/L) | 3.25 ± 0.66 | 4.21 ± 0.75 | 4.261 ± 0.71 | 4.88 ± 0.48 | **0.0025** |
| Platelets (10^9^/L) | 363.33 ± 171.28 | 264.17 ± 149.41 | 316.57 ± 67.57 | 280.83 ± 55.03 | 0.396 |
| Neutrophils (10^9^/L) | 6.83 ± 2.47 | 3.24 ± 0.86 | 5.56 ± 2.41 | 2.96 ± 0.77 | **0.002** |
| Lymphocytes (10^9^/L) | 1.21 ± 0.30 | 0.98 ± 0.43 | 1.260 ± 0.50 | 2.21 ± 0.58 | **0.0009** |
| Monocytes (10^9^/L) | 0.680 ± 0.062 | 0.483 ± 0.300 | 0.600 ± 0.351 | 0.565 ± 0.134 | 0.481 |
| Eosinophils (10^9^/L) | 0.264 ± 0.167 | 0.122 ± 0.106 | 0.447 ± 0.271 | 0.232 ± 0.220 | 0.090 |
| Basophils (10^9^/L) | 0.026 ± 0.015 | 0.018 ± 0.013 | 0.037 ± 0.015 | 0.058 ± 0.029 | **0.010** |
| NRBC (10^9^/L) | 0.200 ± 0.235 | 0.100 ± 0.155 | 0.023 ± 0.040 | 0 | 0.162 |
| Haemoglobin (g/dL) | 9.85 ± 1.65 | 12.25 ± 1.33 | 10.43 ±1.69 | 15.07 ± 1.17 | **<0.0001** |
| HCT (L/L) | 0.294 ± 0.039 | 0.358 ± 0.037 | 0.318 ± 0.035 | 0.425 ± 0.034 | **<0.0001** |
| MCV (fL) | 91.43 ± 7.76 | 86.33 ± 8.14 | 81.43 ± 4.48 | 87.32 ± 2.36 | 0.175 |
| MCH (pg) | 30.60 ± 3.94 | 29.53 ± 2.98 | 26.56 ± 1.70 | 30.95 ± 1.21 | 0.157 |
| MCHC (g/dL) | 33.42 ± 2.07 | 34.18 ± 0.57 | 32.67 ± 1.75 | 35.43 ± 1.09 | 0.050 |
| RDW-SD (fL) | 47.86 ± 8.43 | 42.42 ± 5.68 | 29.40 ±20.25 | 37.62 ± 1.07 | 0.062 |
| RDW-CV (%) | 14.90 ± 1.94 | 13.40 ± 1.13 | 16.13 ± 4.44 | 11.93 ± 0.44 | **0.026** |
| PDW (fL) | 13.02 ± 1.88 | 11.93 ± 2.46 | 9.03 ± 0.32 | 10.54 ± 0.96 | **0.027** |
| MPV (fL) | 10.98 ± 0.98 | 10.50 ± 1.24 | 9.62 ± 0.85 | 9.68 ± 0.53 | **0.044** |
| P-LCR (%) | 32.44 ± 7.27 | 28.65 ± 10.38 | 17.13 ± 2.10 | 21.76 ± 4.20 | **0.031** |
| PCT (%) | 0.432 ± 0.174 | 0.265 ± 0.136 | 0.260 ± 0.056 | 0.280 ± 0.037 | 0.097 |
| WRR | 2.96 ± 0.96 | 1.16 ± 0.15 | 1.25 ± 0.30 | 2.05 ± 0.56 | 0.512 |
| PWR | 39.98 ± 21.10 | 51.60 ± 22.66 | 47.94 ± 10.88 | 37.77 ± 10.57 | 0.969 |
| PNR | 60.26 ± 28.00 | 76.86 ±32.95 | 99.52 ± 26.13 | 60.21 ± 8.73 | 0.975 |
| NWR | 0.745 ± 0.071 | 0.664 ± 0.085 | 0.490 ± 0.060 | 0.645 ± 0.160 | 0.798 |
| NLR | 5.964 ± 2.42 | 4.12 ± 2.60 | 1.37 ± 0.311 | 3.96 ± 2.85 | 0.84 |
| PLR | 354.52 ± 153.76 | 311.47 ±182.55 | 130.97 ± 29.20 | 225.81 ± 137.29 | **0.017** |
| PMR | 596.93 ± 266.68 | 645.93 ± 468.79 | 515.15 ± 126.88 | 665.26 ± 281.79 | 0.969 |
| PRR | 112.77 ± 57.56 | 59.50 ± 27.71 | 58.27 ± 14.20 | 69.64 ± 15.62 | 0.105 |
| MRR | 0.174 ± 0.089 | 0.115 ± 0.067 | 0.118 ± 0.035 | 0.098 ± 0.015 | 0.821 |
| NRR | 1.748 ±1.14 | 0.762 ± 0.102 | 0.612 ± 0.170 | 1.081 ± 0.452 | 0.821 |
| LRR | 0.325 ±0.212 | 0.245 ± 0.142 | 0.457 ± 0.123 | 0.314 ± 0.112 | 0.825 |

HCT – haematocrit; MCV – mean corpuscular volume; MCH – mean corpuscular haemoglobin; MCHC - mean corpuscular haemoglobin concentration; RDW – red cell distribution width; platelet distribution width; MPV – mean platelet volume; PCT – plateletcrit; PLCR – platelet-large cell ratio; NRBC – nucleated red blood cells; WRR – White cell-to-red blood cell-ratio; PWR – platelet-to-white cell-ratio; PNR- platelet-to-neutrophil-ratio; NWR – neutrophil-to-white cell-ratio; NLR – neutrophil-to-lymphocyte-ratio; PLR – platelet-to-lymphocyte-ratio; PMR – platelet-to-monocyte-ratio; PRR – platelet-to-red blood cell-ratio; MRR – monocyte-to-red blood cell-ratio; NRR - neutrophil-to-red blood cell-ratio; LRR - lymphocyte-to-red blood cell-ratio
