# Supplementary material for: COVID-19 induces a hyperactive phenotype in circulating platelets
Source: PLoS Biol. 2021 Feb 17;19(2):e3001109. doi: 10.1371/journal.pbio.3001109 (PMC7920383; doi:10.1371/journal.pbio.3001109)
Supplement: S2 Table — (DOCX) [file pbio.3001109.s004.docx]

**S2 Table**: Mean ADP release and fold changes in ADP release upon platelet stimulation with various agonists.

|  |  | **0.1 U/ml thrombin** | **0.5 U/ml thrombin** | **1 µM U46619** | **6.6 µM U46619** |
| --- | --- | --- | --- | --- | --- |
| **Mean±SD ADP release (pmol/10^6^ platelets)** | sCOV19 | 3.01 ± 1.50 | 3.61 ± 0.75 | 10.71 ± 3.79 | 20.19 ± 4.54 |
|  | nsCOV19 | 3.05 ± 1.13 | 3.56 ± 0.86 | 12.68 ± 4.96 | 19.97 ± 2.08 |
|  | HoC | 0.92 ± 0.14 | 1.07 ± 0.16 | 0.13 ±0.03 | 1.44 ± 0.21 |
|  | HeC | 0.89 ± 0.30 | 1.48 ± 0.46 | 0.44 ± 0.50 | 1.71 ± 0.27 |
| **Fold changes in ADP release** | sCOV19 vs nsCOV19 | 0.99 | 1.01 | 0.84 | 1.01 |
|  | sCOV19 vs HoC | 3.26 | 3.39 | 82.67 | 14.06 |
|  | sCOV19 vs HeC | 3.36 | 2.43 | 24.34 | 11.79 |
|  | nsCOV19 vs HoC | 3.30 | 3.34 | 97.94 | 13.91 |
|  | nsCOV vs HeC | 3.41 | 2.40 | 28.84 | 11.66 |

sCOV19 – severe COVID-19; nsCOV19 – non-severe COVID-19; HoC – hospitalised controls; HeC - healthy controls.
