# Supplementary material for: COVID-19 induces a hyperactive phenotype in circulating platelets
Source: PLoS Biol. 2021 Feb 17;19(2):e3001109. doi: 10.1371/journal.pbio.3001109 (PMC7920383; doi:10.1371/journal.pbio.3001109)
Supplement: Attachment — Submitted filename: Responses to Reviewer Comments.docx [file pbio.3001109.s006.docx]

**REVIEWER 1:**

In this manuscript by Comer and colleagues, they present very timely work on the hyperactivity of platelets in COVID-19.  This is of interest since there is a high degree of thromboembolic disease in COVID-19 and even with anticoagulation, patients still at risk of thrombosis.  In this manuscript the authors demonstrate that patients with COVID-19 have hyperactive circulating platelets and they suggest that this may contribute to the hypercoabulability seen in these patients.

We thank Reviewer 1 for their careful and detailed reading of our manuscript and appreciate the reviewer’s assessment that our work is timely, particularly given the escalation of COVID-19 infection across Europe over the previous weeks. We have now comprehensively addressed all Reviewer 1’s comments and concerns below and any resultant changes to our manuscript are highlighted in the track changes version of our re-submitted manuscript. For convenience page, paragraph and line numbers that refer to new text included in our revised manuscript (following reviewer comments/suggestions) specifically refer to the track changes manuscript.

A few concerns that I have include:

1. A large proportion of the patients with more severe presentations of COVID-19 often present with cardiovascular disease such as hypertension or CAD.  Since these patients are usually placed on aspirin at baseline and since this manuscript is suggesting the platelet activity is linked to disease severity; it begs the question of whether or not any of these patients were on aspirin.  Where there patients who developed covid who were also taking aspirin?  Did those patients have hyperactive platelets?  Did they have increased thrombotic events?

As the reviewer has astutely highlighted, patients with severe COVID-19 may have a background medical history that requires anti-platelet treatment, with aspirin being a widely used therapy. Therefore, due to the established effects of aspirin on platelet function, aspirin therapy in the preceding two weeks was an exclusion criterion in our study and we sincerely apologise that we did not make this sufficiently clear in our original submission. We have amended our methods to make this clearer in our revised submission (retitled *Patient recruitment, blood collection and platelet preparation)* on page 6, paragraph 1, lines 13-18 as follows:

*“Inability to provide informed consent, age younger than 18 years and exposure to anti-platelet agents were contraindications to study inclusion in any group. In particular, the use of aspirin or other antiplatelet agents in the preceding two weeks was an absolute contraindication to inclusion as these agents have direct effects on platelet function and would therefore interfere with the interpretation of results.”*

As aspirin treatment was an exclusion criterion, the suggested analysis to investigate if patients developed COVID while taking aspirin and whether these patients still had hyperactive platelets is unfortunately outside the scope of this study.

2. The authors provide information on the mean platelet volume and platelet reactivity in COVID-19 patients.  Further analysis of MPV and platelet reactivity as it directly relates to development of a thromboembolic event would directly link hyperactive platelets and hypercoagulability.  What was the MPV and platelet activity status in patients with thrombosis and was it different in comparison to those who did not develop clots.

The rationale of our present study was to assess baseline characteristics of platelet activity in COVID-19 patients versus non-COVID-19 patients/controls, hence we specifically recruited patients who had not exhibited a thromboembolic event during hospitalisation. We sincerely apologise that we did not include this information in our original submission. We have now amended our methods in our revised submission (retitled *Patient recruitment, blood collection and platelet preparation)* on page 6, paragraph 1, lines 18-19 as follows:

“*Thromboembolic events during length of hospitalisation were also considered a contraindication to inclusion in the study*”

Consequently, we therefore cannot address Reviewer 1’s comments re platelet hyperreactivity directly relating to the development of a thromboembolic event. We do, however, completely agree with the reviewer’s observation that a deeper analysis of MPV and platelet activation state in patients with thrombosis when compared to non-thrombotic patients, would give great insight into the underlying mechanisms of COVID-19 associated thrombosis/hypercoagulability. International trials are now ongoing that will evaluate the impact of antiplatelet medication on thrombosis risk, which may shed light on this very interesting question, in an RCT design that will be sufficiently powered to address this question (including NCT NCT04368377). Other ongoing trials include evaluation of MPV (NCT04408378; NCT04378829).

3. Although the platelets were hyperactive by the authors description, a functionality component needs to be demonstrated.  Were there changes in platelet aggregation assays?  What about thrombotic assays?

We completely agree with the reviewer that highlighting platelet functionality is important to support our findings of a hyperactive platelet phenotype, therefore, we included the assessment of a basic function of platelets- that is, dense granule secretion upon agonist activation (Figure 2 in manuscript), which importantly has not yet been investigated in another COVID-19 patient cohort. Crucially, we show that SARS-CoV-2 infection induces a 30 to 90-fold increase in platelet dense granule secretion in response to various agonist concentrations, providing unique results regarding the impact of COVID-19 on platelets and their secretory response. In fact, our data suggests that platelets from COVID-19 patients are more responsive to low dose agonist stimulation when compared to control platelets, potentially indicating a reduced activation threshold in all COVID-19 patients. Intriguingly, previous reports have demonstrated similar patterns of enhanced platelet reactivity in response to subthreshold concentrations of agonists in the presence of dengue virus nonstructural protein 1 (1, 2), indicating a potential virus-induced sensitisation of platelets. One of these reports (1) also noted enhanced platelet ADP secretion, akin to our study (Figure 2, main manuscript). As platelet dense granules are packed full of locally-acting autocrine agonists including ADP, ATP and thromboxane A2, which can rapidly amplify platelet activation and trigger additional release of platelet granule contents, our findings provide further evidence that platelets from COVID-19 patients are more reactive, a phenomenon which may contribute to an increased thrombotic risk.

In answering this critique it is important to note that there are four classical platelet activation processes which may be routinely assayed; platelet adhesion, shape change, aggregation and secretion (3). The reviewer specifically mentions the functional assay of platelet aggregation in COVID-19 patients and, indeed, this has previously been assessed with Manne *et al*. showing that platelets in COVID-19 patients displayed an increased aggregatory response (4) and Zaid *et al*. demonstrating that platelet aggregation occurred at suboptimal thrombin concentrations in COVID-19 (5). Therefore, due to this existing data and our limited access to donor blood, we chose not to assess the aggregatory response (*as the volume of blood required to assess multiple agonists at varying concentrations by aggregation was beyond the scope of our ethical approval*) and instead we used our well-published (6, 7), high-throughput (96 well plate) functional assay of dense granule release to assess our COVID-19 cohort. This also enabled us to perform a much wider assessment of platelet agonists at varying concentrations which, to date, has not been investigated in another COVID-19 patient cohort. Our assay is based on detection of dense granule-derived ATP, which reacts with the ATP detecting reagent with subsequent luminescence quantified and this technique has previously been used for functionally evaluating dense granule deficiencies such as storage pool defects or specific defects in platelet degranulation (3, 8).

With regards to the reviewers specifc question re thrombotic assays, calibrated automated thrombography (CAT) or thrombin generation (TG) is a classical method of assessing thrombotic activity in patients. When performed in platelet-poor-plasma (PPP) TG assays give data and insight into the functioning coagulation system whereas, when performed in platelet-rich plasma (PRP), the assay provides information on both the coagulation system and platelet thrombotic activity. White *et al*. (9) recently showed that TG in PPP did not increase in COVID-19 patients admitted to critical care versus non-critical care but that D-dimer and fibrinogen levels (widely regarded as reliable markers of thrombotic activity (10-12)) were increased between cohorts (please note that no control group, healthy or otherwise, were included in this study (9)). We also measured both D-dimer and fibrinogen levels in our study and found them both to be elevated (when compared to normal ranges) in our COVID-19 cohorts indicating increased thrombotic activity (Table 1; main manuscript), in line with current literature (10-12). Although we had not specifically measured TG in either PPP or PRP previously, in response to the reviewer’s comment and for review purposes only, we have now performed a preliminary pilot analysis of TG in PRP according to previously defined protocols (13, 14) in two COVID-19 positive patients (where adequate PRP was available) versus one healthy donor (Figure A).

**(i)	(ii)**

**Figure A. Preliminary data demonstrating that thrombin generation in platelet-rich-plasma may be increased among patients** **infected with COVID-19.** (i) Baseline thrombin generation graph, highlighting parameters measured by calibrated automated thrombography as before (13-15). (ii) peak thrombin generation (nM) in PRP in COVID-19 patients (**Red** n=2; (median ETP 1619 (1538-1700) nm*min ) is increased when compared to healthy donor (**Blue** n=1; 1524nm*min).

We find a small increase in endogenous thrombin potential in PRP (measured as Peak Thrombin in nM) in our two COVID-19 patients in comparison to the single healthy control indicating that there may be increased thrombotic activity in COVID-19 infection (Figure Aii). As Ireland is currently in a second wave of COVID-19 infection, we are currently identifying and recruiting a much larger cohort of patients and controls to enable a more definitive assessment of platelet-dependent hypercoagulability in COVID-19 infection however, this wider investigation is on-going and outside the scope of this current study.

4. The authors discuss agonist-induced ADP release.  Platelets carry a myriad of inflammatory chemokines and also coagulation factors.  What other factors were released from these hyperactive platelets and does a particular factor correlate with disease prognosis?

We thank the reviewer for this truly intriguing observation and agree that platelets carry a wide array of inflammatory chemokines and pro-coagulant factors and that it is of great interest to fully understand platelet-derived secreted factors in COVID-19 patients. In fact, we have studied this important platelet cargo carried in their preformed granules (alpha, dense and lysosomes) for several years (16-22). Therefore, as we had already assessed platelet dense granule secretion (Figure 2 in manuscript) we also quantified circulating levels of two key alpha granular derived factors; the platelet-specific chemokine platelet-factor 4 (PF4) and the soluble version of the cell adhesion molecule P-selectin (sP-selectin) (20, 23). Platelet factor 4 is a powerful soluble effector found only in platelet alpha-granules and is secreted in large quantities upon platelet activation (24, 25). On the other hand, P-selectin is found on the surface of platelet alpha-granular membranes (and on endothelial cell membranes) and post-platelet activation and fusion with the plasma membrane, cleaved platelet P-selectin is widely recognised to circulate in plasma in soluble form (sP-selectin) (26). Both PF4 and sP-selectin have been shown to be released by hyperactive platelets in a number of inflammatory diseases, where they play a role in disease aetiology (27, 28). For example, PF4 has been implicated in atherosclerosis (29, 30), inflammatory bowel disease (27) and arthritis (31) whilst increased sP-selectin levels have been similarly implicated in inflammatory diseases (32), and increased levels of which may be a consequence rather than a cause of disease state (26).

We demonstrated increased PF4 and sP-selectin levels in COVID-19 plasma when compared to healthy and hospitalised controls (Figure 3A & 3B in manuscript). We also found that circulating sP-selectin levels, but not PF4, correlated with disease state between our severe and non-severe COVID-19 cohort. Our findings are incredibly consistent with other literature that has recently demonstrated increased circulating levels of both PF4 and sP-selectin in COVID-19 (4, 5). It is however important to note here that sP-selectin may be also derived from the endothelium (33) and endothelial dysfunction is an observed pathology of COVID-19 (34, 35). Therefore, the observed sP-selectin-based differentiation of COVID-19 cohorts may be due to increased endothelial dysfunction in our severe cohort (Figure 3 in manuscript). As there is currently no defined scoring system to classify COVID-19 severity, akin to for example the expanded disability status scale in Multiple Sclerosis (36), correlation analyses of disease prognosis with the quantity of a particular factor released from hyperactive platelets is unfortunately not possible.

5.  The authors measure soluble P-selectin.  P-selectin can come from activated platelets but also activated endothelial cells.  We know that COVID-19 is associated with enhanced endothelial injury.  The authors should directly measure P-selectin on the platelet surface instead of just soluble P-selectin as a more direct measure of platelet hyperactivity.

We thank reviewer 1 for this insightful comment. As we note above, we absolutely agree with Reviewer 1 that sP-selectin can also be derived from the endothelial membranes (33) and that endothelial dysfunction is an observed pathology of COVID-19 (34, 35). We also agree that direct measurement of P-selectin expression on the activated platelet surface would reinforce our observation of platelet hyperactivity in COVID-19 however, due to restrictions in Ireland (among the harshest in Europe) that were in place during the first COVID-19 lockdown (during which time this study took place), access to a flow cytometer machine in a Biosafety Level 3 laboratory in order to directly measure P-selectin on the platelet surface, was not available to us and this, unfortunately, still remains the case.

We have performed Pearson correlation analyses comparing sP-selectin levels with both platelet counts and PF4 levels (Figure B). These analyses show that our increased sP-selectin levels correlate with platelet count (*r*=0.602, *p*=0.004), consistent with previous studies (37) (Figure Bi). Importantly, we also found a strong and highly significant positive correlation of sP-selectin and PF4 levels (*r*=0.833, *p*<0.0001) (Figure Bii), suggesting that increased levels of both these factors may be platelet-derived.

**Figure B:** **Significant correlation of soluble P-selectin levels with platelet count and PF4 levels.** Pearson correlation analysis (*r)* shows sP-selectin (sCD62P) levels correlate with (i) patients’ platelet count (*r*=0.602, *p*=0.004) and strongly with (ii) PF4 levels (*r*=0.833, *p*<0.0001).

We do understand that although these correlations are strong, they do not replace FACS analysis, therefore, the above figure is for review purposes only.

6. The authors point out that there is no thrombocytosis in patients.  Yet, the TPO levels and MPV are increased which would suggest that the bone marrow is revved up for making platelets.  What is keeping the platelet count from becoming elevated?  We know that platelets are increased in inflammatory conditions, why is it not increased in COVID which is associated with high levels of inflammation?  This especially needs to be explained since the TPO levels are elevated in their study.

As the reviewer states secondary thrombocytosis can occur as a result of inflammatory conditions such as cancer (38, 39), inflammatory bowel disease (40, 41) and bacterial infections (42). In fact, many studies as have shown thrombocytosis to be reactive in nature, caused by an underlying infection or inflammation (43). Interestingly however, thrombocytopenia (a decreased platelet count) is well-recognised to occur in response to viral infections and to occur alongside increased MPV and TPO levels (44). Specifically, thrombocytopenia has been documented in response to SARS-CoV, MERS-CoV and Dengue virus infections (1, 45-47). Indeed, platelets have been found to directly sense and activate in response to SARS-CoV as well as Dengue virus (48, 49). Increased platelet size, measured as mean platelet volume (MPV) is also a sensitive indicator of circulating platelet activity (50-52). Thrombocytopenia is usually associated with a rise in MPV and is suggestive of active turnover of platelet production in bone marrow as a result of platelet consumption (52). Increased MPV is seen with Dengue infection coupled with thrombocytopenia (53); and furthermore, increased TPO has also been documented in Dengue patients again coupled with thrombocytopenia (54). Moreover, in Dengue infection increased platelet activity was shown to be not only associated with thrombocytopenia (55) but it was reported that platelet activity determines the severity of the concomitant thrombocytopenia in Dengue infection (45).

In our COVID-19 cohorts we observed similar patterns of thrombocytopenia, increased MPV and increased TPO comparable to other viral infections discussed above, suggesting as the reviewer states, the bone marrow is primed for platelet production. Our findings also mirror reports in patients with SARS-CoV infection, with the literature showing evidence of thrombocytopenia and increased MPV (47) as well as increased TPO levels (56). In fact, thrombocytopenia has been documented in patients with COVID‐19 and was found to be associated with increased risk of in‐hospital mortality (57-59).The literature on viral infections and other severe infections characterised by critical illness therefore show a pattern of thrombocytopenia coupled with increased MPV and TPO levels (4, 5, 56, 60, 61), and comparable findings have also been reported for patients with SARS-CoV-2 infection.

Viruses have been shown to modulate platelet formation at various stages of their development in the bone marrow. Notably, viruses can alter the levels of circulating cytokines which can cause increased TPO production in the liver (62-64). Moreover, Gibellini et al. found virally (HIV) infected megakaryocytes express less c-Mpl (TPO receptor) on their surface (65). Manne et al. have also shown elevated levels of circulating TPO in COVID-19 patients but critically found that genomic c-Mpl expression was significantly decreased in COVID-19 patients (4), reflecting findings in HIV. Decreased expression of c-Mpl on the surface of platelets and megakaryocytes has been shown to influence circulating TPO levels (66, 67) which may account for the elevated levels of TPO seen in COVID-19.

While the observation of thrombocytopenia and increased TPO maybe counterintuitive as highlighted by the reviewer, it is reflective of the current literature and the precise underlying mechanism remains to be elucidated in both COVID-19 and other viral infections. The most likely explanation is that platelet overproduction and consumption may be simultaneously ongoing. We agree that detailed characterization of this intriguing phenomenon will be valuable.

7. Although there were differences in the hypereactivity of patients in the nonsevere and severe groups, proper controls with patients with equal inflammatory states need to be performed.

We thank the reviewer for highlighting this important point. Recruitment of patients and controls to this study was undertaken during the initial wave of SARS-CoV-2 infections (April and May 2020). During this time much of our hospital capacity was taken up by COVID-19 patients, routine elective care was largely cancelled and vulnerable individuals (such as the elderly or those with chronic medical/inflammatory illnesses) were advised to restrict their social contacts and to limit hospital encounters where possible. Consequently, our approach to recruitment of ‘controls’ was a pragmatic one. Ideally, we would have aimed to recruit patients with influenza or other viral acute respiratory distress syndromes as controls, however unfortunately we did not have access to such patients at the time of recruitment. Therefore, we recruited healthy controls (akin to other published platelet COVID-19 studies (4, 5)), but also, in recognition of the fact that medical illness itself (even in addition to viral infection) likely affects coagulation and platelet activity, we recruited a cohort of hospitalised medical in-patients without COVID-19 to act as a second control group. These medical in-patients were comparable to our COVID-19 cohorts in terms of baseline characteristics (Table 1 in main manuscript).

While it is difficult to predict the course of COVID-19 in the coming months and how this might affect the provision of care to individuals with influenza and similar diseases (and indeed the ability of individuals with influenza to access care in a health system over-burdened with COVID-19), we hope to be in a position in the near future to also explore platelet activity in a patient group with influenza and directly compare platelet function to patients with COVID-19.

8. Do patients with other highly inflammatory conditions also have platelet hypereactivity?  Is this a specific phenomemon on COVID-19.

In short, yes a myriad of highly inflammatory conditions have been reported as exhibiting increased platelet hyperactivity, indicating that this observation is not COVID-19 specific. For example, patients in the febrile and defervescent (68) stages of Dengue virus infection have been shown to have increased P-selectin and phosphatidylserine expression on their platelet surfaces (55). Additionally, it has been previously found that platelet secretion of PF4 is also increased in viral infection (69). Studies of other, non-viral, inflammatory disorders have also reported evidence of platelet hyperactivity including inflammatory arthritis (70), early onset pre-eclampsia (71-73) and Multiple Sclerosis (74). Lastly, inflammatory diseases with which platelets have classically been associated, most notably atherosclerosis (75, 76) (underlying myocardial infarction and ischaemic stroke) also show evidence of platelet hyperactivity.

In fact, thromboinflammation (thrombosis with associated inflammation (77)) is now widely viewed as a key component underlying many disease pathologies and platelets are recognised as a critical crosslinker between the inflammatory, thrombotic and immune (innate and adaptive) processes (48, 78-80). Additionally, anti-platelet drugs, such as aspirin, are commonly used in the treatment of inflammatory conditions (81-84) , including cancer (85, 86), reaffirming the importance of platelets in thromboinflammation. Finally, there are currently 4 ongoing clinical trials investigating the therapeutic efficacy of antiplatelet agents in the treatment of COVID-19-associated thromboinflammation (NCT04391179, NCT04363840, NCT04368377, NCT04365309 (87)), denoting the importance of platelet hyperactivity in this disease.

9.  The authors need to directly link the hyperactivity of platelets to the hypercoagulable state in COVID-19.

Platelet activation and the coagulation cascade are indeed intimately linked with both platelets and coagulation factors contributing to physiologic thrombus formation. The most abundantly expressed platelet glycoprotein, integrin α_IIb_β_3_, for example binds fibrinogen (88). Moreover, platelet phospholipid membranes facilitate the generation of thrombin by providing a negatively charged surface as an assembly site for the pro-thrombinase complex, in addition to thrombin itself being a potent platelet activator (89) that activates platelets through interaction with protease receptors 1 and 4 on the platelet (90). Coagulation Factor V is also contained within platelet α-granules and constitutes about 20% of circulating Factor V (23). Platelets, upon activation, can release anti-coagulant proteins such as tissue factor pathway inhibitor and protease nexin-1 (SERPINE2), which act as a negative feedback loop on the coagulation cascade (91). Platelet-derived extracellular vesicles (EVs) also play a key role in coagulation (92, 93) with tissue factor (TF) being a key procoagulant protein contained in these EVs (94).

Platelets and the coagulation cascade are also linked pathologically. For example, elevated MPV, with larger, more reactive platelets is a predictor for VTE, particularly VTE of unprovoked origin (95). This intrinsic link between platelets and coagulation in pathology is well known with evidence of hyperactivation of platelets in hypercoagulable disorders shown as far back as the 1970’s (96). A hypercoagulable state is accepted as the driver behind thrombosis in antiphospholipid syndrome but a prominent role for platelets has come to light in recent years (97). Platelet-derived P-selectin is known to be associated with atherosclerotic wall changes in the carotid artery in human subjects (98) while sP-selectin is recognised as a biomarker in the diagnosis of deep vein thrombosis alongside D-dimer (99). Platelet-derived coagulation Factor V has also been identified as a critical mediator of arterial thrombosis (100). Platelet EVs are well recognised as contributors to thrombotic disorders due to their procoagulant functions (93, 101, 102). For example, platelet EVs are significantly increased in deep vein thrombosis (99) and have been suggested as a prognostic marker for atherosclerosis (103).

As discussed in a previous response, the rationale of our study was to assess baseline characteristics of platelet activity in COVID-19 patients versus non-COVID-19 patients/controls, hence we specifically recruited patients who had not exhibited a thromboembolic event during hospitalisation. While assessment of thromboembolic events was beyond the scope of the study we have demonstrated strong links between platelet hyperactivity and COVID-19-associated hypercoagulability. We have shown that circulating platelets are both hypersensitive to platelet agonists and hyperactive (Figures 2 and 3 in main manuscript). This is in addition to elevated D-dimer levels (Table 1 in main manuscript), a known marker for increased thrombotic activity, in our COVID-19 cohorts which shows these patients were in a hypercoagulable state compared to our hospital in-patient controls. It is important to note that a recent study showed COVID-19 patients also exhibited increased phosphatidylserine-exposing platelet extracellular vesicles (5), a key component of hypercoagulability mediated by platelets. To further support the evidence of the link between hyperactive platelets and hypercoagulability, we have presented results above (Figure A, for review purposes only) from a small pilot study showing thrombin generation in platelet-rich-plasma is elevated in COVID-19 compared to a healthy control, further linking the observed platelet hyperactivity with hypercoagulation in COVID-19 patients.

Finally, to further link the hyperactivity of platelets to the hypercoagulable state in COVID-19, we have performed a Pearson correlation analysis (Figure C., below; for review purposes only) which shows MPV (a known surrogate for platelet activity (104)) and D-dimer levels (marker of increased thrombotic activity) correlate strongly in COVID-19 patients, further supporting a direct link between platelet hyperactivity and COVID-19-induced hypercoagulability. This is supported by the literature where previous studies show increased MPV is a risk factor for VTE (95, 105).

**Figure C: Significant correlation between COVID patients' MPV and D-Dimer levels.** Pearson correlation analysis (*r)* shows significant positive correlation between their MPV and D-Dimer levels in COVID-19 patients (r=0.792, *p*=0.002).

**REVIEWER 2:**

This is a well written paper that is highly relevant to the current COVID-19 pandemic.  The authors demonstrate differences in routine blood parameters that were associated with disease severity.  Platelets form patients with COVID-19 were more prone to activation, demonstrated by ADP release and soluble markers of activation.  This study is limited by a small sample size as discuss within the manuscript, nonetheless these are interesting observations that could prove to be clinically useful during the current pandemic.
I have some points to address as detailed below.

We thank Reviewer 2 for their kind comments and their considered and detailed reading of our manuscript. We have now comprehensively addressed all Reviewer 2’s comments and concerns below and any resultant changes to our manuscript are highlighted in the ‘track changes’ version of our re-submitted manuscript. For convenience page, paragraph and line numbers that refer to new text included in our revised manuscript (following reviewer comments/suggestions) specifically refer to the track changes manuscript.

What are the in-patient control group, did any of the patients have a history of thrombotic events, have inflammatory diseases/infections?

The inpatient control group consisted of individuals admitted to the care of the acute medical service of our hospital with acute medical illness (lower respiratory tract infection, urinary infection or skin infection) but all had tested negative for SARS-CoV-2. Patients presenting with a major pro-inflammatory/pro-thrombotic state such as recent major surgery/surgical illness, major traumatic injury or active cancer were excluded. None of the patients in the control group had a history of arterial/venous thrombosis.

Thrombosis is a leading source of morbidity and mortality in COVID-19. Numerous investigators have demonstrated that patients with COVID-19 who are admitted to hospital appear to develop thrombosis more frequently than hospitalised patients *without* COVID-19 (106). However, the true magnitude of the difference in thrombotic risk in COVID-19 inpatients in contrast to the general medical inpatient population continues to be a source of significant debate. While we did recruit a cohort of healthy volunteers as a control group in order to investigate differences in platelet activity between healthy individuals and COVID-19 patients, we felt that this comparison would do little to address the dilemmas outlined above (i.e. what is the mechanism and magnitude of thrombotic risk between COVID-19 and non-COVID in-patients). We therefore chose to include a separate control cohort of general medical inpatients as described above (but with careful adherence to our exclusion criteria in order to ensure that patients with confounding factors such as severe liver/kidney disease, cancer etc. would not be included). The magnitude of difference in platelet activity observed between this inpatient cohort and the COVID-19 group provides much more compelling evidence in support of the hypothesis that COVID-19 is associated with increased platelet activity in hospitalised COVID-19 (in contrast to general medical patients) than would have been provided by the comparison with young, healthy volunteer group in isolation. We feel this observation is of significant clinical relevance.

We have amended our methods section entitled *Patient recruitment, blood collection and platelet preparation* on page 6, parapgraph 1, lines 19-23 as follows;

*The hospitalised control group consisted of individuals admitted to our hospital with acute medical illness (lower respiratory tract infection, urinary infection or skin infection) and without history of arterial/venous thrombosis. Patients presenting with a major pro-inflammatory/pro-thrombotic state such as recent major surgery/surgical illness, major traumatic injury or active cancer were excluded.*

The healthy controls were not on aspirin/anti-platelet agents - what about the in-patient group?

As Reviewer 2 points out, our healthy control cohort were not on aspirin/anti-platelet agents (we also discuss this above in response to a critique from Reviewer 1). In fact, all our patient cohorts (in-patient control group or COVID-19 groups) were recruited with the criterion that none had been administered aspirin/anti-platelet agents in the preceding two weeks as this was considered an absolute contraindication to study inclusion. To ensure absolute clarity in our patient recruitment process in relation to this point, we have amended our methods section *Patient recruitment, blood collection and platelet preparation* on page 6, paragraph 1, lines 13-18 as follows:.

*“Inability to provide informed consent, age younger than 18 years and exposure to anti-platelet agents were contraindications to study inclusion in any group. In particular, the use of aspirin or other antiplatelet agents in the preceding two weeks was an absolute contraindication to inclusion as these agents have direct effects on platelet function and would therefore interfere with the interpretation of results.”*

Unless I missed it, no reference is made in the text is made to supplementary Table 1

We thank the reviewer for highlighting our previous error and sincerely apologise for the omission of a reference to Supplementary Table 1 in the text of our manuscript. We have now included reference to it on page 9, paragraph 2, lines 19-20 of our manuscript.

The methods require some more details and some clarification.

*       Please state number of patients recruited per group

We thank the reviewer for this comment. We have amended our manuscript to include patient recruitment numbers in the methods section entitled *Patient recruitment, blood collection and platelet preparation (page 6, paragraph 1, lines 6 and 7*. Furthermore, in response to a comment from the Academic Editor we have also included a flow diagram (new Supplementary Fig.1) detailing the methodology of our study, which also conveniently includes the numbers of patients included in each experiment/analysis.

*       ACD Vacutainer solution A/B?

The vacutainer solution used in this study was ACD-A. We thank the reviewer for bringing this omission to our attention and have amended our manuscript accordingly on page 6, paragraph 2, lines 28 and 30.

*       Presumably the pH was adjusted with a buffer, eg. modified tyrodes or hepes etc during washing.  The methods state it was with ACD?

Reviewer 2 is correct in stating that the pH of platelet-rich-plasma (PRP) was adjusted using ACD-A buffer to approximately pH 6.5. Platelets, once isolated from PRP, were then resuspended in a modified Tyrode’s buffer whose pH had also been adjusted, in this case to pH 7.4, using ACD-A buffer prior to platelet resuspension. This is outlined in our manuscript on page 6, paragraph 2, lines 29-30

*       What buffer were the isolated platelets resuspended in?  In the platelet ATP secretion assay platelet washing is described?  If this was not different to the above this information only needs to be stated once

We thank the reviewer for this comment and we sincerely apologise for any previous omission in our platelet isolation and preparation processes. We wish to clarify that platelets, once isolated from PRP, were resuspended in a modified Tyrode’s buffer (130 mM NaCl, 9 mM NaHCO3, 10 mM Tris-HCl, 10 mM Trisodium citrate, 3 mM KCl, 0.81 mM KH2PO4, 9 mM MgCl2 x 6H2O) whose pH had been adjusted to pH 7.4, using ACD-A buffer prior to platelet resuspension. Therefore, we have now included the following text in the *Patient recruitment, blood collection and platelet preparation* section on pages 6 and 7, paragraphs 2 and 1, lines 31-32 and 1-4, respectively, of the revised manuscript:

*“Isolated platelets from patient groups and healthy controls were washed using a modified Tyrode’s buffer (130 mM NaCl, 9 mM NaHCO3, 10 mM Tris-HCl, 10 mM Trisodium citrate, 3 mM KCl, 0.81 mM KH2PO4, 9 mM MgCl2 x 6H2O; pH 7.4 with ACD) followed by centrifugation (600 xg for 10 min at RT). Platelets were again resuspended in modified Tyrode’s buffer and incubated at 37^o^C.”*

We also fully agree with the reviewer that this information should not be repeated and have thus appropriately deleted the first paragraph of *Platelet ATP secretion assay* section of the methods. We thank the reviewer again for bringing these points to our attention and for giving us the opportunity to rectify and amend.

Are data mean +/- IQR? Please state in figure legends.

We thank the reviewer for this comment and sincerely apologise for omitting an explanation of the boxplot representation of the data in our previously submitted figure legends. Indeed, our boxplots represent data median (line inside the box) and the interquartile range (outline of the box) together with data maximum & data minimum (whiskers) and individual observations (individual point shapes: diamond, triangle, square).

Accordingly, we have now added the following explanation to each figure legend in our revised main manuscript (pages 10, 12 and 13) as well as in supplementary figure 2: *“**Boxplots represent the data median (line inside the box) and the interquartile range (IQR; outline of the box) together with data maximum and data minimum (whiskers) and individual observations.”*

Can the authors comment on the fibrinogen levels - in other studies these are reportedly raised on admission.

We agree with Reviewer 2 that fibrinogen levels have been reported to be elevated in other studies in patients with COVID-19(52). In fact, we have also demonstrated elevated fibrinogen levels in our Covid-19 positive study cohort, but as we did not include the reference ranges for fibrinogen in our manuscript, this fact may not have been previously apparent. We, therefore, thank the reviewer for bringing this to our attention and we have accordingly amended **Table 1 (**pages 9 and 10) to now include these reference ranges, which highlight the presence of elevated fibrinogen levels in our COVID-19 patients. We have also included reference ranges for PT, aPTT and D-dimer.

Typo page 14 - appear to be associated idwith significant morbidity

We thank the reviewer for identifying this typographical error, which has now been corrected in our revised manuscript.

Comments from the Academic Editor:

It is not clear enough why what measurement was done in which patient. There needs to be more information available on this, and that information is relevant to understanding where the data come from. I am not saying that there need to be more measurement done, but it has to be justified why the measures have been performed in a selection of the patients. A simple flowchart explaining the flow between fig 1, 2, and 3 would already help a lot.

We thank the Academic Editor for their comment and for kindly conducting the review process with their thorough reading of our manuscript. We greatly welcome the opportunity to clarify specific measurements performed and based on the Academic Editor’s excellent suggestion we have now created a flowchart showing the experimental and methodological approach adopted in our study. The flowchart has been included in our revised manuscript as supplementary figure 1 with reference to the flowchart on page 8, line 20 of the main manuscript. We also include the flowchart here (Figure D, below) for ease of reference.

**Figure D:** Flow Diagram of the methodology employed to assess platelet activity in COVID-19. Severe COVID-19 (blue; n=34), non-severe COVID-19 (red; n=20) and hospitalised controls (grey; n=20) were recruited for the current study from the Mater Misericordiae University Hospital (gender distribution denoted by male and female symbols). Full blood count analysis was performed for these 74 patients. Further in-depth analyses (platelet secretion assays and platelet activity markers) were performed on a randomly selected subset of recruited patients; severe COVID-19 (blue; n=6), non-severe COVID-19 (red; n=6) and hospitalised controls (grey; n=6) in addition to healthy controls (yellow; n=6).

We again thank the Academic Editor for their incredibly helpful suggestion and believe the addition of this flowchart will add significantly to our manuscript and will aid the PLoS Biology readership in fully appreciating the work presented therein.

**References:**

1.	Chao CH, Wu WC, Lai YC, Tsai PJ, Perng GC, Lin YS, et al. Dengue virus nonstructural protein 1 activates platelets via Toll-like receptor 4, leading to thrombocytopenia and hemorrhage. PLoS pathogens. 2019;15(4):e1007625.

2.	Quirino-Teixeira AC, Rozini SV, Barbosa-Lima G, Coelho DR, Carneiro PH, Mohana-Borges R, et al. Inflammatory signaling in dengue-infected platelets requires translation and secretion of nonstructural protein 1. Blood Adv. 2020;4(9):2018-31.

3.	Pakala R, Waksman R. Currently available methods for platelet function analysis: advantages and disadvantages. Cardiovascular revascularization medicine : including molecular interventions. 2011;12(5):312-22.

4.	Manne BK, Denorme F, Middleton EA, Portier I, Rowley JW, Stubben CJ, et al. Platelet Gene Expression and Function in COVID-19 Patients. Blood. 2020.

5.	Zaid Y, Puhm F, Allaeys I, Naya A, Oudghiri M, Khalki L, et al. Platelets can contain SARS-CoV-2 RNA and are hyperactivated in COVID-19. medRxiv. 2020:2020.06.23.20137596.

6.	Lombardi F, De Chaumont C, Shields DC, Moran N. Platelet signalling networks: pathway perturbation demonstrates differential sensitivity of ADP secretion and fibrinogen binding. Platelets. 2012;23(1):17-25.

7.	Mitrugno A, Williams D, Kerrigan SW, Moran N. A novel and essential role for FcgammaRIIa in cancer cell-induced platelet activation. Blood. 2014;123(2):249-60.

8.	Pai M, Wang G, Moffat KA, Liu Y, Seecharan J, Webert K, et al. Diagnostic usefulness of a lumi-aggregometer adenosine triphosphate release assay for the assessment of platelet function disorders. American journal of clinical pathology. 2011;136(3):350-8.

9.	White D, MacDonald S, Edwards T, Bridgeman C, Hayman M, Sharp M, et al. Evaluation of COVID-19 coagulopathy; laboratory characterization using thrombin generation and nonconventional haemostasis assays. International Journal of Laboratory Hematology. 2020;n/a(n/a).

10.	Garcia-Olivé I, Sintes H, Radua J, Abad Capa J, Rosell A. D-dimer in patients infected with COVID-19 and suspected pulmonary embolism. Respir Med. 2020;169:106023-.

11.	Lippi G, Favaloro EJ. D-dimer is Associated with Severity of Coronavirus Disease 2019: A Pooled Analysis. Thrombosis and haemostasis. 2020;120(5):876-8.

12.	Zhang L, Yan X, Fan Q, Liu H, Liu X, Liu Z, et al. D-dimer levels on admission to predict in-hospital mortality in patients with Covid-19. J Thromb Haemost. 2020;18(6):1324-9.

13.	Egan K, van Geffen JP, Ma H, Kevane B, Lennon A, Allen S, et al. Effect of platelet-derived β-thromboglobulins on coagulation. Thromb Res. 2017;154:7-15.

14.	Parsons ME, O'Connell K, Allen S, Egan K, Szklanna PB, McGuigan C, et al. Thrombin generation correlates with disease duration in multiple sclerosis (MS): Novel insights into the MS-associated prothrombotic state. Multiple sclerosis journal - experimental, translational and clinical. 2017;3(4):2055217317747624.

15.	Egan K, O'Connor H, Kevane B, Malone F, Lennon A, Al Zadjali A, et al. Elevated plasma TFPI activity causes attenuated TF-dependent thrombin generation in early onset preeclampsia. Thrombosis and haemostasis. 2017;117(8):1549-57.

16.	Coppinger JA, Cagney G, Toomey S, Kislinger T, Belton O, McRedmond JP, et al. Characterization of the proteins released from activated platelets leads to localization of novel platelet proteins in human atherosclerotic lesions. Blood. 2004;103(6):2096-104.

17.	Coppinger JA, O'Connor R, Wynne K, Flanagan M, Sullivan M, Maguire PB, et al. Moderation of the platelet releasate response by aspirin. Blood. 2007;109(11):4786-92.

18.	Grande R, Dovizio M, Marcone S, Szklanna PB, Bruno A, Ebhardt HA, et al. Platelet-Derived Microparticles From Obese Individuals: Characterization of Number, Size, Proteomics, and Crosstalk With Cancer and Endothelial Cells. Frontiers in pharmacology. 2019;10:7.

19.	Maguire PB, Parsons ME, Szklanna PB, Zdanyte M, Münzer P, Chatterjee M, et al. Comparative Platelet Releasate Proteomic Profiling of Acute Coronary Syndrome versus Stable Coronary Artery Disease. Frontiers in cardiovascular medicine. 2020;7:101.

20.	Parsons MEM, Szklanna PB, Guerrero JA, Wynne K, Dervin F, O'Connell K, et al. Platelet Releasate Proteome Profiling Reveals a Core Set of Proteins with Low Variance between Healthy Adults. Proteomics. 2018;18(15):e1800219.

21.	Szklanna PB, Parsons ME, Wynne K, O'Connor H, Egan K, Allen S, et al. The Platelet Releasate is Altered in Human Pregnancy. Proteomics Clinical applications. 2019;13(3):e1800162.

22.	Szklanna PB, Wynne K, Nolan M, Egan K, Áinle FN, Maguire PB. Comparative proteomic analysis of trophoblast cell models reveals their differential phenotypes, potential uses, and limitations. Proteomics. 2017;17(10):e1700037.

23.	Blair P, Flaumenhaft R. Platelet alpha-granules: basic biology and clinical correlates. Blood Rev. 2009;23(4):177-89.

24.	Shuman MA, Levine SP. Thrombin generation and secretion of platelet Factor 4 during blood clotting. The Journal of clinical investigation. 1978;61(4):1102-6.

25.	Ginsberg M, Taylor L, Painter R. The mechanism of thrombin-induced platelet factor 4 secretion. Blood. 1980;55(4):661-8.

26.	Panicker SR, Mehta-D’souza P, Zhang N, Klopocki AG, Shao B, McEver RP. Circulating soluble P-selectin must dimerize to promote inflammation and coagulation in mice. Blood. 2017;130(2):181-91.

27.	Ye L, Zhang Y-P, Yu N, Jia Y-X, Wan S-J, Wang F-Y. Serum platelet factor 4 is a reliable activity parameter in adult patients with inflammatory bowel disease: A pilot study. Medicine (Baltimore). 2017;96(11):e6323-e.

28.	Turkoz Y, Evereklioglu C, Ozkiriş A, Mistik S, Borlu M, Ozerol IH, et al. Serum levels of soluble P-selectin are increased and associated with disease activity in patients with Behçet's syndrome. Mediators of inflammation. 2005;2005(4):237-41.

29.	Sachais BS, Turrentine T, Dawicki McKenna JM, Rux AH, Rader D, Kowalska MA. Elimination of platelet factor 4 (PF4) from platelets reduces atherosclerosis in C57Bl/6 and apoE-/- mice. Thrombosis and haemostasis. 2007;98(5):1108-13.

30.	Koenen RR, von Hundelshausen P, Nesmelova IV, Zernecke A, Liehn EA, Sarabi A, et al. Disrupting functional interactions between platelet chemokines inhibits atherosclerosis in hyperlipidemic mice. Nature Medicine. 2009;15(1):97-103.

31.	Shi G, Field DJ, Long X, Mickelsen D, Ko K-a, Ture S, et al. Platelet factor 4 mediates vascular smooth muscle cell injury responses. Blood. 2013;121(21):4417-27.

32.	Woollard KJ, Chin-Dusting J. P-selectin antagonism in inflammatory disease. Current pharmaceutical design. 2010;16(37):4113-8.

33.	Lorenzon P, Vecile E, Nardon E, Ferrero E, Harlan JM, Tedesco F, et al. Endothelial cell E- and P-selectin and vascular cell adhesion molecule-1 function as signaling receptors. J Cell Biol. 1998;142(5):1381-91.

34.	Ackermann M, Verleden SE, Kuehnel M, Haverich A, Welte T, Laenger F, et al. Pulmonary Vascular Endothelialitis, Thrombosis, and Angiogenesis in Covid-19. New England Journal of Medicine. 2020.

35.	Varga Z, Flammer AJ, Steiger P, Haberecker M, Andermatt R, Zinkernagel AS, et al. Endothelial cell infection and endotheliitis in COVID-19. Lancet (London, England). 2020;395(10234):1417-8.

36.	Inojosa H, Schriefer D, Ziemssen T. Clinical outcome measures in multiple sclerosis: A review. Autoimmunity reviews. 2020;19(5):102512.

37.	Semenov AV, Romanov YA, Loktionova SA, Tikhomirov OY, Khachikian MV, Vasil'ev SA, et al. Production of soluble P-selectin by platelets and endothelial cells. Biochemistry Biokhimiia. 1999;64(11):1326-35.

38.	Bailey SE, Ukoumunne OC, Shephard EA, Hamilton W. Clinical relevance of thrombocytosis in primary care: a prospective cohort study of cancer incidence using English electronic medical records and cancer registry data. Br J Gen Pract. 2017;67(659):e405-e13.

39.	Voutsadakis IA. Thrombocytosis as a prognostic marker in gastrointestinal cancers. World J Gastrointest Oncol. 2014;6(2):34-40.

40.	Yan SLS, Russell J, Harris NR, Senchenkova EY, Yildirim A, Granger DN. Platelet abnormalities during colonic inflammation. Inflamm Bowel Dis. 2013;19(6):1245-53.

41.	Voudoukis E, Karmiris K, Koutroubakis IE. Multipotent role of platelets in inflammatory bowel diseases: a clinical approach. World J Gastroenterol. 2014;20(12):3180-90.

42.	Finsterer J, Kotzailias N. Thrombocytosis under ciprofloxacin and tazobactam/piperacillin. Platelets. 2003;14(5):329-31.

43.	Rose SR, Petersen NJ, Gardner TJ, Hamill RJ, Trautner BW. Etiology of thrombocytosis in a general medicine population: analysis of 801 cases with emphasis on infectious causes. J Clin Med Res. 2012;4(6):415-23.

44.	Assinger A. Platelets and infection - an emerging role of platelets in viral infection. Frontiers in immunology. 2014;5:649-.

45.	Ojha A, Nandi D, Batra H, Singhal R, Annarapu GK, Bhattacharyya S, et al. Platelet activation determines the severity of thrombocytopenia in dengue infection. Scientific reports. 2017;7:41697.

46.	Assiri A, Al-Tawfiq JA, Al-Rabeeah AA, Al-Rabiah FA, Al-Hajjar S, Al-Barrak A, et al. Epidemiological, demographic, and clinical characteristics of 47 cases of Middle East respiratory syndrome coronavirus disease from Saudi Arabia: a descriptive study. Lancet Infect Dis. 2013;13(9):752-61.

47.	Chen R-F, Chang J-C, Yeh W-T, Lee C-H, Liu J-W, Eng H-L, et al. Role of vascular cell adhesion molecules and leukocyte apoptosis in the lymphopenia and thrombocytopenia of patients with severe acute respiratory syndrome (SARS). Microbes Infect. 2006;8(1):122-7.

48.	Guo L, Rondina MT. The Era of Thromboinflammation: Platelets Are Dynamic Sensors and Effector Cells During Infectious Diseases. Frontiers in Immunology. 2019;10(2204).

49.	Giannis D, Ziogas IA, Gianni P. Coagulation disorders in coronavirus infected patients: COVID-19, SARS-CoV-1, MERS-CoV and lessons from the past. J Clin Virol. 2020;127:104362-.

50.	Bath PMW, Butterworth RJ. Platelet size: measurement, physiology and vascular disease. Blood Coagulation & Fibrinolysis. 1996;7(2):157-61.

51.	Gasparyan AY, Ayvazyan L, Mikhailidis DP, Kitas GD. Mean platelet volume: a link between thrombosis and inflammation? Current pharmaceutical design. 2011;17(1):47-58.

52.	Handtke S, Thiele T. Large and small platelets-(When) do they differ? J Thromb Haemost. 2020.

53.	Sharma K, Yadav A. Association of Mean Platelet Volume with Severity, Serology & Treatment Outcome in Dengue Fever: Prognostic Utility. J Clin Diagn Res. 2015;9(11):EC01-EC3.

54.	Fatima H, Riaz M, Mahmood Z, Yousaf F, Shahid M. Dengue viral infection deteriorates vitamin D3, K, thrombopoietin, and angiotensinogen levels in humans. European Journal of Inflammation. 2018;16:2058739218791100.

55.	Hottz ED, Oliveira MF, Nunes PCG, Nogueira RMR, Valls-de-Souza R, Da Poian AT, et al. Dengue induces platelet activation, mitochondrial dysfunction and cell death through mechanisms that involve DC-SIGN and caspases. Journal of thrombosis and haemostasis : JTH. 2013;11(5):951-62.

56.	Yang M, Ng MHL, Li CK, Chan PKS, Liu C, Ye JY, et al. Thrombopoietin levels increased in patients with severe acute respiratory syndrome. Thrombosis research. 2008;122(4):473-7.

57.	Guan W-j, Ni Z-y, Hu Y, Liang W-h, Ou C-q, He J-x, et al. Clinical Characteristics of Coronavirus Disease 2019 in China. New England Journal of Medicine. 2020;382(18):1708-20.

58.	Chen N, Zhou M, Dong X, Qu J, Gong F, Han Y, et al. Epidemiological and clinical characteristics of 99 cases of 2019 novel coronavirus pneumonia in Wuhan, China: a descriptive study. Lancet (London, England). 2020;395(10223):507-13.

59.	Yang X, Yang Q, Wang Y, Wu Y, Xu J, Yu Y, et al. Thrombocytopenia and its association with mortality in patients with COVID-19. Journal of Thrombosis and Haemostasis.n/a(n/a).

60.	Lupia E, Goffi A, Bosco O, Montrucchio G. Thrombopoietin as biomarker and mediator of cardiovascular damage in critical diseases. Mediators Inflamm. 2012;2012:390892.

61.	Zakynthinos SG, Papanikolaou S, Theodoridis T, Zakynthinos EG, Christopoulou-Kokkinou V, Katsaris G, et al. Sepsis severity is the major determinant of circulating thrombopoietin levels in septic patients. Crit Care Med. 2004;32(4):1004-10.

62.	Metcalf Pate KA, Lyons CE, Dorsey JL, Queen SE, Adams RJ, Morrell CN, et al. TGFβ-Mediated Downregulation of Thrombopoietin Is Associated With Platelet Decline in Asymptomatic SIV Infection. Journal of acquired immune deficiency syndromes (1999). 2014;65(5):510-6.

63.	Isomura H, Yoshida M, Namba H, Fujiwara N, Ohuchi R, Uno F, et al. Suppressive effects of human herpesvirus-6 on thrombopoietin-inducible megakaryocytic colony formation in vitro. The Journal of general virology. 2000;81(Pt 3):663-73.

64.	Gonelli A, Mirandola P, Grill V, Secchiero P, Zauli G. Human herpesvirus 7 infection impairs the survival/differentiation of megakaryocytic cells. Haematologica. 2002;87(11):1223-5.

65.	Gibellini D, Clò A, Morini S, Miserocchi A, Ponti C, Re MC. Effects of human immunodeficiency virus on the erythrocyte and megakaryocyte lineages. World J Virol. 2013;2(2):91-101.

66.	Fielder PJ, Gurney AL, Stefanich E, Marian M, Moore MW, Carver-Moore K, et al. Regulation of thrombopoietin levels by c-mpl-mediated binding to platelets. Blood. 1996;87(6):2154-61.

67.	Horikawa Y, Matsumura I, Hashimoto K, Shiraga M, Kosugi S, Tadokoro S, et al. Markedly reduced expression of platelet c-mpl receptor in essential thrombocythemia. Blood. 1997;90(10):4031-8.

68.	Costa VV, Fagundes CT, Souza DG, Teixeira MM. Inflammatory and innate immune responses in dengue infection: protection versus disease induction. The American journal of pathology. 2013;182(6):1950-61.

69.	Srichaikul T, Nimmannitya S, Sripaisarn T, Kamolsilpa M, Pulgate C. Platelet function during the acute phase of dengue hemorrhagic fever. The Southeast Asian journal of tropical medicine and public health. 1989;20(1):19-25.

70.	Mac Mullan PA, Peace AJ, Madigan AM, Tedesco AF, Kenny D, McCarthy GM. Platelet hyper-reactivity in active inflammatory arthritis is unique to the adenosine diphosphate pathway: a novel finding and potential therapeutic target. Rheumatology (Oxford, England). 2010;49(2):240-5.

71.	Janes SL, Goodall AH. Flow cytometric detection of circulating activated platelets and platelet hyper-responsiveness in pre-eclampsia and pregnancy. Clinical science (London, England : 1979). 1994;86(6):731-9.

72.	Jakobsen C, Larsen JB, Fuglsang J, Hvas A-M. Platelet function in preeclampsia – a systematic review and meta-analysis. Platelets. 2019;30(5):549-62.

73.	Monteith C, Egan K, O’Connor H, Maguire P, Kevane B, Szklanna PB, et al. Early onset preeclampsia is associated with an elevated mean platelet volume (MPV) and a greater rise in MPV from time of booking compared with pregnant controls: results of the CAPE study. Journal of Perinatal Medicine. 2018;46(9):1010.

74.	Saluk-Bijak J, Dziedzic A, Bijak M. Pro-Thrombotic Activity of Blood Platelets in Multiple Sclerosis. Cells. 2019;8(2):110.

75.	Mezger M, Nording H, Sauter R, Graf T, Heim C, von Bubnoff N, et al. Platelets and Immune Responses During Thromboinflammation. Frontiers in immunology. 2019;10:1731-.

76.	Ross R. Atherosclerosis--an inflammatory disease. The New England journal of medicine. 1999;340(2):115-26.

77.	Jackson SP, Darbousset R, Schoenwaelder SM. Thromboinflammation: challenges of therapeutically targeting coagulation and other host defense mechanisms. Blood. 2019;133(9):906-18.

78.	Rayes J, Bourne JH, Brill A, Watson SP. The dual role of platelet-innate immune cell interactions in thrombo-inflammation. Research and Practice in Thrombosis and Haemostasis. 2020;4(1):23-35.

79.	Gawaz M, Langer H, May AE. Platelets in inflammation and atherogenesis. J Clin Invest. 2005;115(12):3378-84.

80.	Morrell CN, Aggrey AA, Chapman LM, Modjeski KL. Emerging roles for platelets as immune and inflammatory cells. Blood. 2014;123(18):2759-67.

81.	DAVÌ G, SANTILLI F. Unveiling the inflammatory face of antiplatelet drugs. Journal of Thrombosis and Haemostasis. 2006;4(10):2137-9.

82.	Müller KA, Chatterjee M, Rath D, Geisler T. Platelets, inflammation and anti-inflammatory effects of antiplatelet drugs in ACS and CAD. Thrombosis and haemostasis. 2015;114(3):498-518.

83.	Pitchford SC. Novel uses for anti-platelet agents as anti-inflammatory drugs. Br J Pharmacol. 2007;152(7):987-1002.

84.	Mora S, Manson JE. Aspirin for Primary Prevention of Atherosclerotic Cardiovascular Disease: Advances in Diagnosis and Treatment. JAMA Internal Medicine. 2016;176(8):1195-204.

85.	Dovizio M, Bruno A, Tacconelli S, Patrignani P. Mode of Action of Aspirin as a Chemopreventive Agent. In: Chan AT, Detering E, editors. Prospects for Chemoprevention of Colorectal Neoplasia: Emerging Role of Anti-Inflammatory Drugs. Berlin, Heidelberg: Springer Berlin Heidelberg; 2013. p. 39-65.

86.	Patrignani P, Patrono C. Aspirin, platelet inhibition and cancer prevention. Platelets. 2018;29(8):779-85.

87.	Mitchell WB. Thromboinflammation in COVID-19 acute lung injury. Paediatr Respir Rev. 2020;35:20-4.

88.	Bennett JS. Structure and function of the platelet integrin alphaIIbbeta3. The Journal of clinical investigation. 2005;115(12):3363-9.

89.	Wood JP, Silveira JR, Maille NM, Haynes LM, Tracy PB. Prothrombin activation on the activated platelet surface optimizes expression of procoagulant activity. Blood. 2011;117(5):1710-8.

90.	Duvernay M, Young S, Gailani D, Schoenecker J, Hamm HE. Protease-activated receptor (PAR) 1 and PAR4 differentially regulate factor V expression from human platelets. Mol Pharmacol. 2013;83(4):781-92.

91.	Heemskerk JWM, Mattheij NJA, Cosemans JMEM. Platelet-based coagulation: different populations, different functions. Journal of Thrombosis and Haemostasis. 2013;11(1):2-16.

92.	Owens AP, 3rd, Mackman N. Microparticles in hemostasis and thrombosis. Circulation research. 2011;108(10):1284-97.

93.	Italiano JE, Jr., Mairuhu ATA, Flaumenhaft R. Clinical relevance of microparticles from platelets and megakaryocytes. Curr Opin Hematol. 2010;17(6):578-84.

94.	Zwicker JI. Tissue factor-bearing microparticles and cancer. Seminars in thrombosis and hemostasis. 2008;34(2):195-8.

95.	Braekkan SK, Mathiesen EB, Njølstad I, Wilsgaard T, Størmer J, Hansen JB. Mean platelet volume is a risk factor for venous thromboembolism: the Tromsø Study, Tromsø, Norway. J Thromb Haemost. 2010;8(1):157-62.

96.	Wu KK, Barnes RW, Hoak JC. Platelet hyperaggregability in idiopathic recurrent deep vein thrombosis. Circulation. 1976;53(4):687-91.

97.	Baroni G, Banzato A, Bison E, Denas G, Zoppellaro G, Pengo V. The role of platelets in antiphospholipid syndrome. Platelets. 2017;28(8):762-6.

98.	Koyama H, Maeno T, Fukumoto S, Shoji T, Yamane T, Yokoyama H, et al. Platelet P-Selectin Expression Is Associated With Atherosclerotic Wall Thickness in Carotid Artery in Humans. Circulation. 2003;108(5):524-9.

99.	Ramacciotti E, Blackburn S, Hawley AE, Vandy F, Ballard-Lipka N, Stabler C, et al. Evaluation of soluble P-selectin as a marker for the diagnosis of deep venous thrombosis. Clin Appl Thromb Hemost. 2011;17(4):425-31.

100.	Ren M, Li R, Chen N, Pang N, Li Y, Deng X, et al. Platelet-Derived Factor V Is a Critical Mediator of Arterial Thrombosis. Journal of the American Heart Association. 2017;6(7):e006345.

101.	Nomura S, Shimizu M. Clinical significance of procoagulant microparticles. Journal of Intensive Care. 2015;3(1):2.

102.	Lipets EN, Antonova OA, Shustova ON, Losenkova KV, Mazurov AV, Ataullakhanov FI. Use of Thrombodynamics for revealing the participation of platelet, erythrocyte, endothelial, and monocyte microparticles in coagulation activation and propagation. PloS one. 2020;15(5):e0227932.

103.	Boulanger CM, Amabile N, Tedgui A. Circulating microparticles: a potential prognostic marker for atherosclerotic vascular disease. Hypertension (Dallas, Tex : 1979). 2006;48(2):180-6.

104.	Park Y, Schoene N, Harris W. Mean platelet volume as an indicator of platelet activation: methodological issues. Platelets. 2002;13(5-6):301-6.

105.	Rupa-Matysek J, Gil L, Barańska M, Dytfeld D, Komarnicki M. Mean platelet volume as a predictive marker for venous thromboembolism in patients treated for Hodgkin lymphoma. Oncotarget [Internet]. 2018 2018/04//; 9(30):[21190-200 pp.].

106.	Middeldorp S, Coppens M, van Haaps TF, Foppen M, Vlaar AP, Muller MCA, et al. Incidence of venous thromboembolism in hospitalized patients with COVID-19. J Thromb Haemost. 2020.
